# The StcE metalloprotease of enterohaemorrhagic Escherichia coli reduces the inner mucus layer and promotes adherence to human colonic epithelium *ex vivo*

**DOI:** 10.1111/cmi.12717

**Published:** 2017-02-15

**Authors:** Claire L. Hews, Seav‐Ly Tran, Udo Wegmann, Bernard Brett, Alistair D.S. Walsham, Devon Kavanaugh, Nicole J. Ward, Nathalie Juge, Stephanie Schüller

**Affiliations:** ^1^ Norwich Medical SchoolUniversity of East AngliaNorwichUK; ^2^ Gut Health and Food Safety ProgrammeInstitute of Food ResearchNorwichUK; ^3^ Department of GastroenterologyNorfolk and Norwich University HospitalNorwichUK; ^4^ School of Biological SciencesUniversity of East AngliaNorwichUK; ^5^ Department of GastroenterologyJames Paget University HospitalGreat YarmouthUK

**Keywords:** adherence, colonic epithelium, EHEC, IVOC, metalloprotease, mucus

## Abstract

Enterohaemorrhagic Escherichia coli (EHEC) is a major foodborne pathogen and tightly adheres to human colonic epithelium by forming attaching/effacing lesions. To reach the epithelial surface, EHEC must penetrate the thick mucus layer protecting the colonic epithelium. In this study, we investigated how EHEC interacts with the intestinal mucus layer using mucin‐producing LS174T colon carcinoma cells and human colonic mucosal biopsies. The level of EHEC binding and attaching/effacing lesion formation in LS174T cells was higher compared to mucin‐deficient colon carcinoma cell lines, and initial adherence was independent of the presence of flagellin, Escherichia coli common pilus, or long polar fimbriae. Although EHEC infection did not affect gene expression of secreted mucins, it resulted in reduced MUC2 glycoprotein levels. This effect was dependent on the catalytic activity of the secreted metalloprotease StcE, which reduced the inner mucus layer and thereby promoted EHEC access and binding to the epithelium *in vitro* and *ex vivo*. Given the lack of efficient therapies against EHEC infection, StcE may represent a suitable target for future treatment and prevention strategies.

## 1 INTRODUCTION

Enterohaemorrhagic Escherichia coli (EHEC) is a major foodborne pathogen in the developed world and affects mainly young children and the elderly (Croxen et al., 2013). Most infections are caused by serotype O157:H7 strains (Gould et al., 2013). In addition to causing diarrhoea and haemorrhagic colitis, EHEC infection can result in life‐threatening haemolytic uraemic syndrome (HUS), which is the main cause of acute kidney failure in children in western countries and can lead to lifelong renal and neurological impairment (Trachtman, Austin, Lewinski, & Stahl, 2012). Notably, there is currently no specific treatment against EHEC infection and HUS, and the use of antibiotics is not recommended due to increased risk of developing HUS (Freedman et al*.*, 2016).

After entering the human gut, EHEC binds to the intestinal epithelium by adhesins such as fimbriae, pili or flagella (McWilliams & Torres, 2014). This initial recognition step is then followed by the formation of firm attaching/effacing (A/E) lesions mediated by a type III secretion system. In particular, the bacterial translocated intimin receptor is injected into the host cell and becomes exposed on the cell membrane. Subsequently, the EHEC outer membrane protein intimin binds to translocated intimin receptor leading to intimate bacterial attachment and initiating further signalling events within the host cell, which ultimately result in actin polymerisation and pedestal formation (Stevens & Frankel, 2014). Although EHEC A/E lesions have not been observed clinically, *ex vivo* culture of human intestinal biopsies indicates EHEC A/E lesion formation in the distal small intestine and colon (Chong et al., 2007; Lewis, Cook, Tighe, & Schüller 2015).

Before adhering to the intestinal epithelium, EHEC must penetrate the mucus layer, which acts as a physicochemical barrier and protects the underlying epithelium from pathogens and foreign antigens. In the colon, the mucus layer is around 400 μm thick and formed of two layers (Johansson et al., 2014; McGuckin, Lindén, Sutton, & Florin 2011). Whereas the inner layer is dense, firmly attached to the epithelium, and virtually free of bacteria, the outer layer is loose, easily penetrable, and densely colonised by the gut microbiota (Johansson et al., 2008). The mucus layer is comprised of mucin glycoproteins secreted by epithelial goblet cells (McGuckin et al., 2011). Around 20 mucins have been identified so far with the majority bound to the cell surface forming the glycocalyx. In contrast, gel‐forming mucin glycoproteins are secreted from goblet cell granules and oligomerize into complex macromolecular structures incorporating water and thereby forming the inner and outer mucus layer (Juge, 2012; McGuckin et al., 2011). In the human intestine, MUC2 is the major secreted mucin of the mucus layer (Johansson et al., 2008).

Although the interaction of EHEC with the intestinal epithelium has been intensely studied, its relationship with the mucus layer remains largely unknown. In this study, we have investigated EHEC binding and its effect on the mucus layer in mucus‐producing human intestinal epithelial cell lines and mucosal biopsy samples.

## 2 RESULTS

### 2.1 EHEC adherence to mucus‐producing and mucus‐deficient intestinal epithelial cells

**Figure 1:**
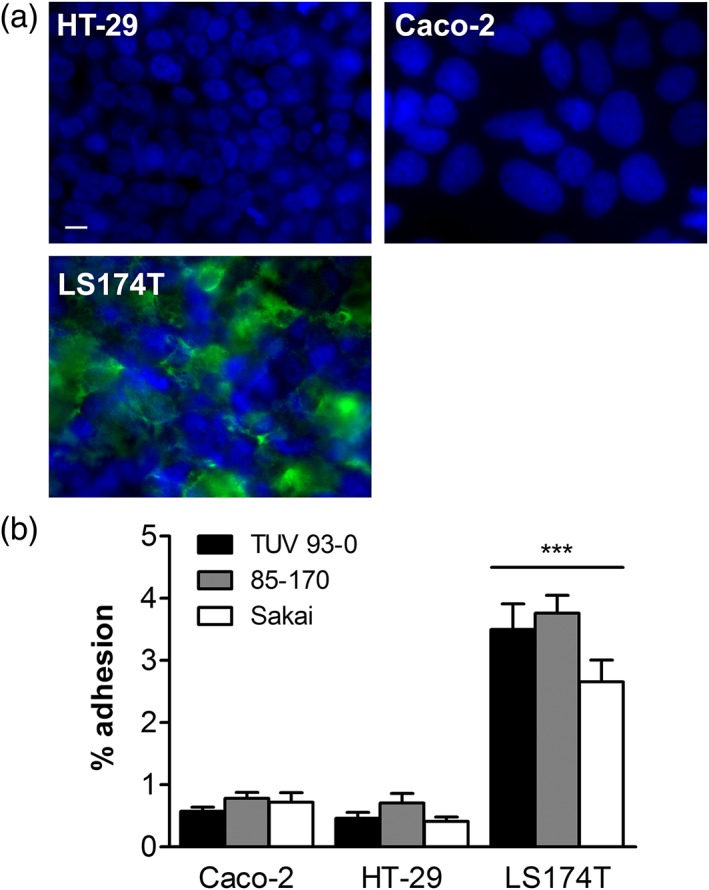
EHEC binding to mucus‐producing LS174T and mucus‐deficient HT‐29 and Caco‐2 cells. (a) Immunofluorescence staining for MUC2 (green) and cell nuclei (blue). Bar =10 μm. (b) Adherence of EHEC TUV 93–0, 85–170 and Sakai after 1 hr of infection. Adhesion was determined by counting colony‐forming units and is expressed as percentage of cell‐bound bacteria relative to the inoculum. ***p < 0.001 versus Caco‐2 and HT‐29 cells

To determine EHEC binding to different colon carcinoma cell lines, colonocyte‐derived HT‐29 and Caco‐2 and goblet cell‐derived LS174T cells were chosen. As shown in Figure 1a, only LS174T cells produced MUC2, whereas no specific staining could be detected for HT‐29 and Caco‐2 cells. Interestingly, binding of all EHEC strains tested (TUV 93‐0, 85‐170, and Sakai) was significantly higher in LS174T cells compared to Caco‐2 and HT‐29 cells after 1 hr of infection (Figure 1b).

**Figure 2:**
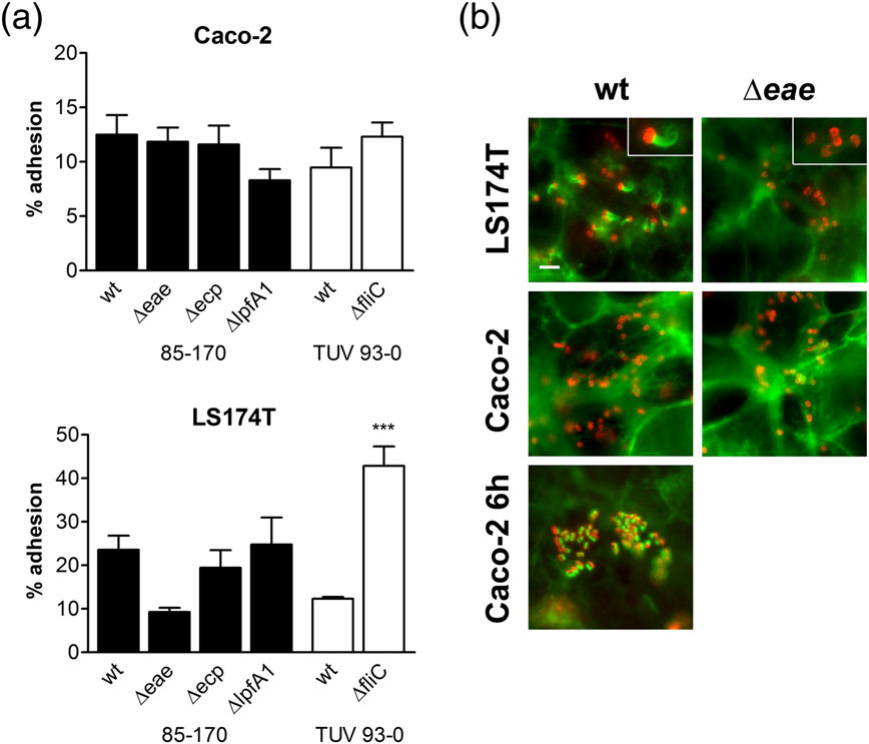
Involvement of EHEC adhesins in binding to Caco‐2 and LS174T cells. (a) Adherence of wild‐type (wt) and adhesin‐deficient EHEC strains after 3 hr of infection. Adhesion was determined by colony‐forming unit counting and is expressed as percentage of cell‐bound bacteria relative to the inoculum. ***p < 0.001 versus wt. (b) Fluorescent actin staining to identify A/E lesion formation. Caco‐2 and LS174T cells were infected with EHEC 85‐170 wt or Δ*eae* for 3 or 6 hr (Caco‐2 for 6 hr) and stained for actin (green) and E. coli (red). Inserts in top right corner show enlarged image areas containing EHEC bacteria with and without actin pedestals (LS174T wt and Δ*eae*, respectively). Bar = 5 μm

We next examined which adhesins were involved in binding to mucus‐deficient Caco‐2 and mucus‐producing LS174T cells. Infections were performed with isogenic EHEC deletion mutants in intimin (Δ*eae*), E. coli common pilus (Δ*ecp*), long polar fimbriae (Δ*lpfA1*), and flagellin (Δ*fliC*). As no significant differences in binding were detected after 1 hr of infection (Figure S1), the incubation period was prolonged to 3 hr to allow sufficient time for expression of adherence factors. Whereas none of the adhesins tested significantly affected EHEC binding to Caco‐2 cells, increased binding to LS174T cells was observed in the absence of flagellin (Figure 2a). In addition, the intimin‐negative mutant showed reduced binding to LS174T cells, although this did not reach significance. Subsequent immunofluorescence staining to detect actin pedestals demonstrated EHEC A/E lesion formation in LS174T but not in Caco‐2 cells after 3 hr of infection. As expected, EHEC Δ*eae* did not form A/E lesions in either cell line (Figure 2b). No pedestal formation was observed in LS174T or Caco‐2 cells after 1 hr (data not shown), and wild‐type EHEC demonstrated actin recruitment in Caco‐2 cells after 6 hr of infection (Figure 2b).

### 2.2 Influence of EHEC infection on mucus production

**Figure 3:**
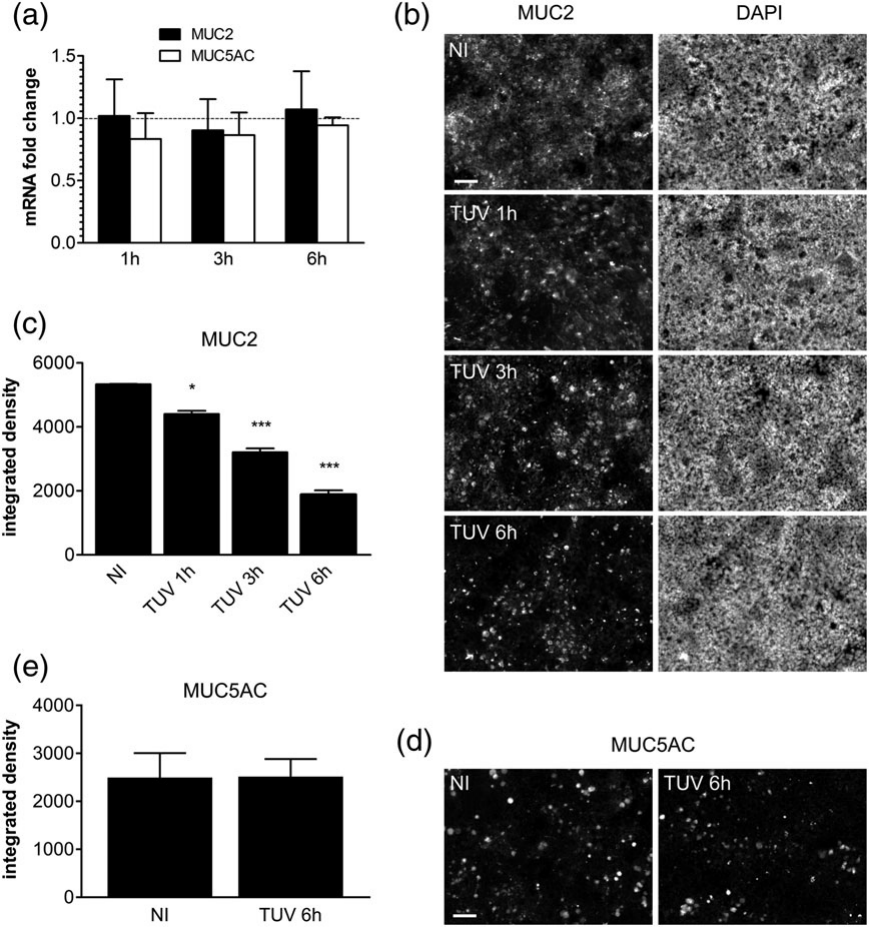
Influence of EHEC infection on mucin expression. LS174T cells were infected with TUV 93‐0 or left non‐infected (NI) for up to 6 hr. (a) MUC2 and MUC5AC gene expression was determined by qPCR, and mRNA levels in infected cells are indicated as fold changes relative to NI controls. (b) MUC2 and (d) MUC5AC protein expression was evaluated by immunofluorescence staining. (b) Cell nuclei were counterstained with DAPI, and images are shown as separate monochrome panels. Bars =50 μm. (c) MUC2 and (e) MUC5AC staining was quantified by integrated density measurement. *p < 0.05, ***p < 0.001 versus NI

In order to determine the effect of EHEC infection on mucus expression, LS174T cells were incubated with EHEC for up to 6 hr, and gene expression of the two major secreted mucins, MUC2 and MUC5AC, was examined by quantitative reverse transcription polymerase chain reaction (qPCR). No significant changes were observed in EHEC‐infected versus non‐infected cells (Figure 3a). In contrast, immunofluorescence staining demonstrated significantly decreased MUC2 protein levels after EHEC infection (Figure 3b,c). Counterstaining of cell nuclei with 4',6‐diamidino‐2‐phenylindole (DAPI) confirmed that this was not due to cell detachment (Figure 3b). In addition, MUC5AC was only produced by a small subset of cells, and staining intensity was not influenced by EHEC infection (Figure 3d,e).

### 2.3 StcE decreases MUC2 protein levels and enhances EHEC binding to colonic epithelium

**Figure 4:**
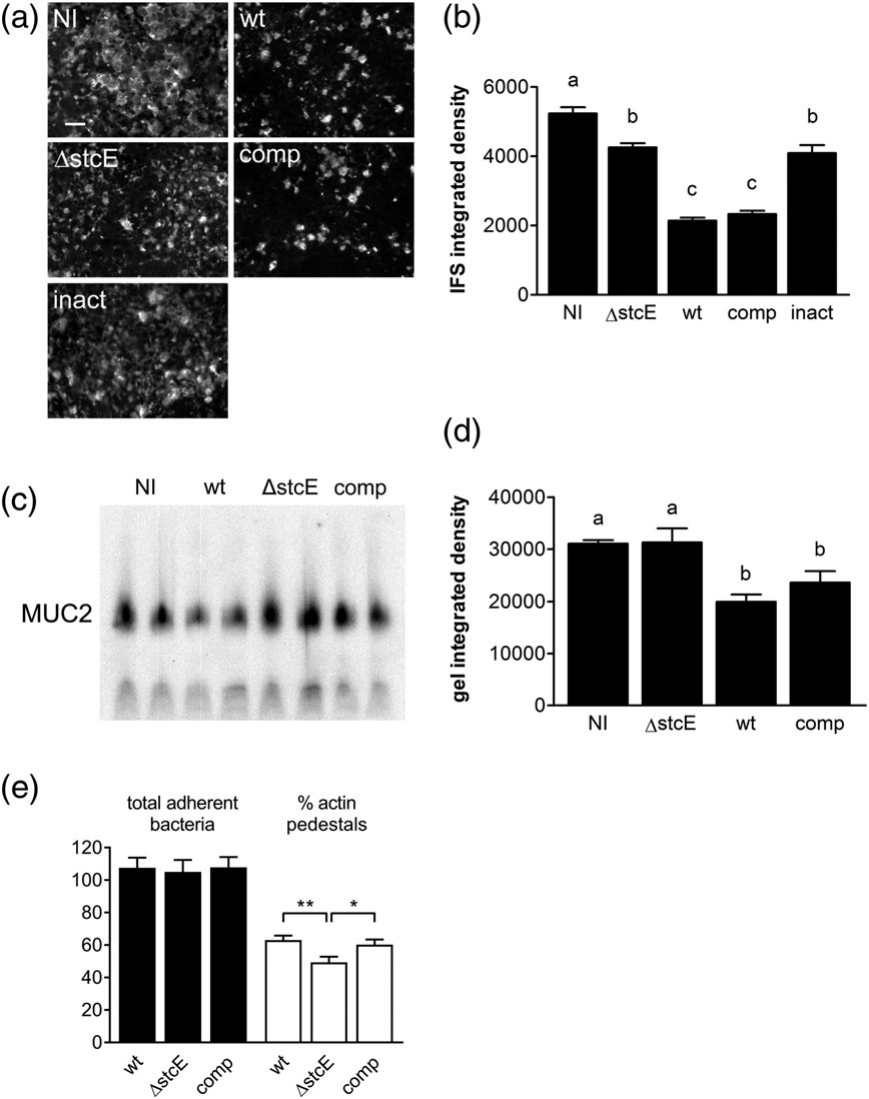
EHEC StcE reduces mucin levels and promotes A/E lesion formation on LS174T cells. Cells were infected with wild‐type TUV 93‐0 (wt), an isogenic *stcE* mutant (ΔstcE), ΔstcE complemented with wild‐type (comp), or catalytically inactive StcE (inact), or left non‐infected (NI) for (a–d) 6 hr or (e) 3 hr. (a) MUC2 expression was determined by immunofluorescence staining, bar = 5 μm or (c) agarose gel electrophoresis and Western blotting and quantified by integrated density measurement (b and d, respectively). Means with different letters are significantly different (*p* < 0.001 for b, *p* < 0.01 for d). *n* = 2 in duplicate for c and d. (e) A/E lesion formation was assessed by immunofluorescence staining and expressed as percentage of bacteria associated with actin pedestals relative to the total number of adherent bacteria, **p < 0.01, *p < 0.05

Previous studies have identified a secreted EHEC metalloprotease (StcE) with mucinase activity for human saliva (Grys, Siegel, Lathem, & Welch, 2005). In order to investigate whether StcE affected intestinal MUC2 levels during EHEC infection, we generated an isogenic *stcE* deletion mutant in strain TUV 93‐0 by Lambda Red recombination. As shown in Figure 4a and b, deletion of *stcE* significantly impaired reduction of MUC2 levels in EHEC‐infected LS174T cells. This was restored to wild‐type levels after complementation with StcE (Figure 4a,b). In contrast, complementation with catalytically inactive StcE (E447D) did not exhibit any effect, and MUC2 levels were comparable to those of the deletion mutant (Figure 4a,b). In addition to immunofluorescence staining, StcE‐dependent MUC2 reduction was confirmed by sodium dodecyl sulfate (SDS)‐agarose gel electrophoresis of cell lysates and subsequent Western blotting with MUC2‐specific antibodies (Figure 4c,d). We further determined whether decreased MUC2 levels facilitated EHEC access and binding to the epithelium and quantified the number of total adherent bacteria (binding to mucus layer and epithelium) and bacteria associated with actin pedestals (binding to epithelium only). Whereas similar numbers of wild‐type, Δ*stcE* and complemented EHEC were associated with LS174T monolayers, a significant reduction in actin pedestal formation was observed with the Δ*stcE* mutant (Figure 4e).

**Figure 5:**
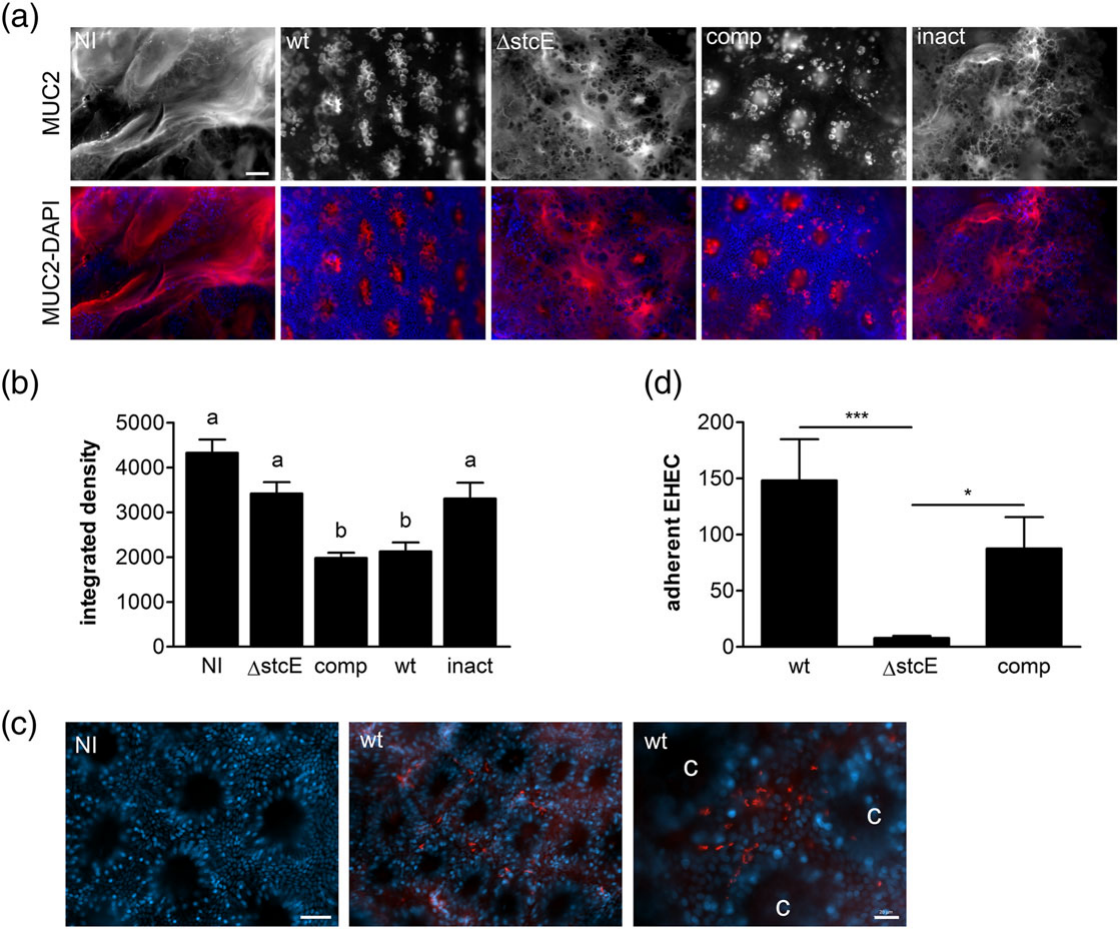
StcE diminishes the inner mucus layer and enhances EHEC adherence to human colonic biopsy epithelium. Tissue samples from the transverse colon were infected with wild‐type TUV 93‐0 (wt), an *stcE* mutant (ΔstcE), ΔstcE complemented with wild‐type (comp), or catalytically inactive StcE (inact), or left non‐infected (NI) for 8 hr. (a) Immunofluorescence staining of the inner mucus layer with anti‐MUC2 (red) and counterstaining of cell nuclei with DAPI (blue). Shown are separate monochrome images of MUC2 staining and merged colour images of both fluorescence channels (MUC2‐DAPI). Bar =50 μm. (b) Quantification of MUC2 staining by integrated density measurement. Means with different letters are significantly different (*p* < 0.001). (c) Immunofluorescence staining of non‐infected (NI) and wild‐type EHEC‐infected biopsies (wt) showing EHEC bacteria (red) and cell nuclei (blue). Shown are merged colour images of both fluorescence channels. c = crypt; bars = 20 μm. (d) Quantification of adherent bacteria per mm^2^ surface area. ***p < 0.001, *p < 0.05. *n* = 4 in triplicate

To confirm the effect of StcE on mucus production and EHEC adherence in a biologically relevant model, we performed *in vitro* organ culture (IVOC) of human colonic biopsy samples (Lewis et al., 2015). Whereas the epithelium was covered by a firmly adherent inner mucus layer in non‐infected tissue samples, diminished MUC2 levels and exposure of underlying crypt‐associated goblet cells was evident after 8 hr of EHEC infection (Figure 5a,b). In contrast, incubation with EHEC Δ*stcE* resulted only in marginal disruption of the mucus layer leading to the appearance of “dark holes” devoid of MUC2 staining. Attenuated mucus reduction by EHEC Δ*stcE* was restored to wild‐type levels after complementation with wild‐type but not catalytically inactive StcE (Figure 5a,b). To further determine the impact of decreased mucus levels on EHEC adherence to the biopsy epithelium, we removed the mucus layer after the IVOC and quantified adherent EHEC by immunofluorescence staining. As shown in Figure 5c, EHEC bacteria were predominantly attached to the intercrypt surface epithelium, and no adhering bacteria were detected in non‐infected control samples. Quantification of adherent bacteria revealed a significant decrease in epithelial binding of EHEC Δ*stcE* compared to the wild‐type and complemented strain (Figure 5d).

## 3 DISCUSSION

EHEC is a colonic pathogen which binds tightly to human intestinal epithelium by forming A/E lesions (Lewis et al., 2015). To do so, the bacteria must first interact with and penetrate the thick mucus layer overlying the colonic epithelium (Johansson et al., 2014; McGuckin et al., 2011). How this happens is still largely unknown due to a paucity of suitable animal models (Ritchie, 2014). In addition, most human cell line models used to study EHEC infection (e.g. T84, Caco‐2, and HT‐29 colon carcinoma cells) are devoid of a MUC2‐containing mucus layer (Navabi, McGuckin, & Lindén 2013; van Klinken et al., 1996; Walsham et al., 2016). Using goblet cell‐like LS174T colon carcinoma cells which produce MUC2 glycoproteins (van Klinken et al., 1996), we found that EHEC O157:H7 prototype strains adhered better to this cell line compared to mucus‐deficient Caco‐2 and HT‐29 cells. A similar phenotype has recently been reported for the related A/E pathogen enteropathogenic E. coli, which demonstrated increased binding to LS174T versus HT‐29 cells (Walsham et al., 2016). In addition, adhesion of Salmonella enterica to mucus‐producing HT‐29 MTX cells was significantly increased compared to mucus‐deficient HT‐29 and Caco‐2 cells (Gagnon, Zihler Berner, Chervet, Chassard, & Lacroix, 2013). Although many different adhesins have been identified for EHEC, there is very limited information on EHEC binding to mucus (McWilliams & Torres, 2014). Previous studies have shown binding of EHEC H7 flagella to isolated bovine colonic mucus and mucin from bovine submaxillary glands and porcine stomach (Erdem, Avelino, Xicohtencatl‐Cortes, & Girón, 2007). Here, we showed increased adhesion of a *fliC* EHEC deletion mutant to LS174T cells compared to the wild‐type strain. This result may be explained by upregulation of other colonisation factors in the *fliC* mutant as previously shown for Pseudomonas aeruginosa, where deletion of flagellin resulted in induction of the type III secretion system (Soscia, Hachani, Bernadac, Filloux, & Bleves, 2007). Furthermore, deletion of long polar fimbriae enhanced EHEC adherence to human intestinal explants suggesting cross talk between bacterial adhesion systems (Fitzhenry et al., 2006). Alternatively, the lack of motility associated with these mutants may lead to enhanced binding or retention in the mucus layer (Rossez, Wolfson, Holmes, Gally, & Holden, 2015). It was further noted that EHEC A/E lesion formation in LS174T cells occurred earlier than in Caco‐2 cells, thereby explaining the observed dependency on intimin for adherence to this cell line. Whether enhanced EHEC binding and A/E lesion formation in LS174T cells is ultimately linked to mucus production or associated with expression of other cell surface receptors remains to be established. In addition, the role of other EHEC adhesins in binding to human intestinal mucus should be investigated.

In the second part of this study, we investigated the influence of EHEC infection on mucus production. Previous reports on the murine A/E pathogen Citrobacter rodentium demonstrated dynamic changes in mucus thickness during infection (Gustafsson et al., 2013). During the acute phase, mucin expression and goblet cell numbers were reduced in the distal colon, and this was dependent on the host adaptive immune response (Bergstrom et al., 2008; Lindén, Florin, & McGuckin, 2008). In contrast, increased mucus secretion at later stages of infection promoted expulsion of bacteria into the gut lumen and clearance of infection (Bergstrom et al., 2010; Gustafsson et al., 2013). The protective role of the mucus layer during Citrobacter infection was further demonstrated by studies in Muc2‐deficient mice, which exhibited increased weight loss and mortality due to elevated pathogen burden at the mucosal surface (Bergstrom et al., 2010). Similar to Citrobacter, infection with atypical but not typical enteropathogenic E. coli increased mucus production in rabbit ileal loops and mucin‐producing HT‐29 MTX cells, which promoted bacterial growth in the absence of luminal flow (Vieira et al., 2010).

Apart from MUC2, LS174T cells also secrete MUC5AC, which is usually expressed in the stomach but also associated with colon cancer (Bartman et al., 1999; van Klinken et al., 1996). In our studies, no changes in MUC2 and MUC5AC gene expression were detected in EHEC‐infected LS174T cells. This is in contrast to previous studies demonstrating increased MUC2 gene expression in HT‐29 cells infected with EHEC (Xue et al., 2014). This discrepancy is likely related to the difference in cell line models, and as HT‐29 cells do not express MUC2 glycoproteins (Huet et al., 1995; Walsham et al., 2016), the relevance of this finding remains questionable.

In contrast to the gene expression results, MUC2 protein levels were significantly decreased in EHEC‐infected LS174T cells. This is in agreement with a recent study reporting reduction of the MUC2‐containing inner mucus layer in EHEC‐infected colonic organoids (In et al., 2016). One likely explanation for this phenotype is the production of mucus‐degrading enzymes, which has been reported in enteropathogens (McGuckin et al., 2011). Shigella flexneri and enteroaggregative E. coli secrete the mucin‐degrading serine protease Pic (Henderson, Czeczulin, Eslava, Noriega, & Nataro, 1999), and *Vibrio cholerae* produces the mucinolytic metalloproteases Hap and TagA (Silva, Pham, & Benitez, 2003; Szabady, Yanta, Halladin, Schofield, & Welch, 2011). Interestingly, the latter has high sequence homology to the StcE zinc metalloprotease from EHEC, which is located on the pO157 virulence plasmid and secreted by a type II secretion system (Lathem et al., 2002). StcE has been shown to modulate the host immune response by cleaving C1 esterase inhibitor and CD43 and CD45 neutrophil glycoproteins (Lathem et al., 2002; Szabady, Lokuta, Walters, Huttenlocher, & Welch, 2009) and also reduces the viscosity of human saliva by degrading mucin 7 and glycoprotein 340 (Grys et al., 2005). However, recombinant StcE did not show any activity against purified human MUC2 (Grys, Walters, & Welch, 2006), and infection of human colonic organoids with an *stcE* mutant only resulted in a slight decrease in mucus reduction compared to the wild‐type strain (In *et al.*, 2016). Here, we found that deletion of *stcE* significantly diminished reduction of mucin levels during EHEC infection of LS174T cells. As catalytic inactivation of StcE had the same effect, it is likely that StcE lowers mucin levels by MUC2 degradation. However, other mechanisms related to mucin glycoprotein synthesis and secretion cannot be excluded at this stage. Diminished mucus reduction in Δ*stcE*‐infected cells was accompanied by a decrease in A/E lesion formation despite adhesion levels comparable to the wild‐type strain. Our findings confirm previous results in HEp‐2 cells demonstrating reduced actin pedestal formation but not overall adherence by Δ*stcE* versus wild‐type EHEC (Grys *et al.*, 2005). HEp‐2 cells do not secrete a mucus layer, and the authors suggested that StcE promotes intimate adherence by cleaving cell surface mucins and thereby exposing host cell receptors. To evaluate our findings in a physiologically relevant setting, we used IVOC of human colonic biopsies to investigate the impact of StcE on mucus production and EHEC adherence *ex vivo*. Previous studies from our laboratory have shown that EHEC binds to human colonic biopsy epithelium by forming A/E lesions (Lewis et al., 2015). Similar to our results in LS174T cells, reduction of the inner MUC2‐containing mucus layer in IVOC was dependent on the presence of catalytically active StcE. However, lack of StcE caused a more pronounced effect on EHEC binding than in the cell line model with an almost complete loss of adherence to the colonic epithelium. It is likely that infection kinetics, environmental cues or host response may fine‐tune expression and/or activity of StcE and other EHEC mucinases, and this may explain the differences in StcE‐mediated mucin reduction observed in IVOC and colonic organoids (In et al., 2016).

Notably, some decrease in mucus levels independent of StcE was evident in LS174T cells and colonic biopsies, suggesting the activity of other mucinolytic enzymes during EHEC infection. Apart from StcE, another type II‐secreted zinc metalloprotease has recently been identified in intestinal and extraintestinal E. coli strains (Nesta et al., 2014). Although SslE facilitates mucus penetration of extraintestinal E. coli in HT‐29 MTX cells and enhances bacterial adhesion to the epithelial surface (Valeri et al., 2015), this protein is rarely detected in EHEC and therefore unlikely to contribute to its pathogenesis (Feng et al., 2007; Nesta et al., 2014).

In summary, our studies have shown that the EHEC‐secreted metalloprotease StcE reduces mucus levels and promotes bacterial adherence to human colonic epithelium *in vitro* and *ex vivo*. Given the lack of effective prevention and treatment strategies, StcE may represent a suitable target for drug and vaccine development against EHEC infection in the human gut.

## 4 Experimental procedures

### 4.1 Bacterial culture

EHEC strains used in this study are listed in Table 1. Bacteria were cultured on Luria‐Bertani (LB) agar or as standing overnight cultures in LB broth at 37 °C unless otherwise stated. Where indicated, mutant strains were grown in the presence of 50 μg/ml kanamycin or 30 μg/ml chloramphenicol (Table 1).

**Table 1:** Bacterial strains

Strain name	Resistance	Reference
TUV 93‐0		A. Donohue‐Rolfe, Tufts University, US
TUV 93‐0 Δ*fliC*	Kan	Erdem et al., 2007
TUV 93‐0 Δ*stcE*	Kan	This study
TUV 93‐0 Δ*stcE* comp	Kan, Cm	This study
TUV 93‐0 Δ*stcE* inact	Kan, Cm	This study
85‐170		Tzipori et al., 1987
85‐170 Δ*eae*		Fitzhenry et al., 2002
85‐170 Δ*ecp*	Kan	Rendón et al., 2007
85‐170 Δ*lpfA1*	Cm	Fitzhenry et al., 2006

### 4.2 Cell culture

HT‐29 (ECACC 91072201), Caco‐2 (ECACC 86010202), and LS174T human colon carcinoma cells (ECACC 87060401) were cultured in Dulbecco's Modified Eagle's Medium (DMEM, Sigma) supplemented with 10% foetal calf serum, 4mM L‐glutamine and 1× nonessential amino acids (Sigma). Cell lines were grown at 37 °C in a 5% CO_2_ atmosphere.

### 4.3 Infection of cell lines

Cells were seeded at a density of 10^5^ (LS174T, Caco‐2) or 1.2 × 10^5^ cells per well (HT‐29) into 24‐well plates and grown for 7 days to reach full confluence. For immunofluorescence staining, cells were grown on glass coverslips. For infection, supplemented DMEM was replaced with plain DMEM medium, and 20 μl bacterial overnight culture (approximately 10^7^ bacteria) were added to each well. Infected cells were incubated at 37 °C in a 5% CO_2_ atmosphere for up to 6 hr. Medium was exchanged after 3 hr to prevent bacterial overgrowth and acidification. At the end of infection, cells were washed three times with phosphate‐buffered saline (PBS) to remove non‐adherent bacteria and processed according to further applications.

### 4.4 Quantification of adherence

Infected cells were lysed with 1% Triton X‐100 in PBS for 15 min. Cell lysates were serially diluted in PBS, and appropriate dilutions were plated on LB agar. Plates were incubated overnight, and colony‐forming units were counted the next day.

### 4.5 IVOC of endoscopic biopsies

This study was performed with approval from the University of East Anglia Faculty of Medicine and Health Ethics Committee (ref 2010/11‐030). All samples were registered with the Norwich Biorepository (NRES ref 08/h0304/85 + 5). Biopsy samples from the transverse colon were obtained with informed consent during colonoscopy of adult patients. Samples were taken from macroscopically normal areas, transported to the laboratory in IVOC medium and processed within the next hour. IVOC was performed as described previously (Lewis et al., 2015). Briefly, biopsies were mounted on foam supports in 12‐well plates and incubated with 25 μl bacterial overnight culture (approximately 10^7^ bacteria). Samples were incubated for 8 hr on a rocking platform at 37 °C in a 5% CO_2_ atmosphere. At the end of the experiment, tissues were either immediately fixed for mucin staining or washed vigorously in PBS to remove the mucus layer and evaluate bacterial adhesion to the epithelium.

### 4.6 Immunofluorescence staining

Cells and biopsy samples were fixed in 3.7% formaldehyde/PBS for 20 min at room temperature or in Carnoy's fixative (60% dry ethanol, 30% chloroform, and 10% glacial acetic acid) overnight at 4 °C for mucin staining. Samples were permeabilised with 0.1% Triton X‐100/PBS if required and were blocked with 0.5% bovine serum albumine (BSA)/PBS for 20 min. Coverslips and tissues were sequentially incubated with primary antibodies (anti‐MUC2 and anti‐MUC5AC from Santa Cruz, anti‐E. coli from Abcam) for 1 hr, followed by incubation in Alexa Fluor 488‐ or Alexa Fluor 568‐conjugated secondary antibodies (Life Technologies) for 30 min. Cell nuclei and filamentous actin were counterstained with DAPI (Roche) and Fluorescein isothiocyanate‐conjugated phalloidin (Sigma) for 30 min, respectively. Cell monolayers and biopsy samples were mounted with Vectashield mounting medium (Vector Labs) and analysed using an Axio Imager M2 motorised fluorescence microscope (Zeiss). MUC2 fluorescence staining was measured by taking images from 10 random fields of view (20× magnification) and determining the integrated density with ImageJ software (https://imagej.nih.gov/ij/). EHEC actin pedestal formation on LS174T cells was quantified by taking images from 10 random fields of view (63× magnification) and counting total bacteria and bacteria associated with actin pedestals. EHEC colonisation of colonic biopsies was evaluated by counting adherent bacteria in a surface area of 1 mm^2^.

### 4.7 SDS‐agarose gel electrophoresis and Western blotting

Confluent LS174T cell monolayers were lysed in ice‐cold lysis buffer (1% Triton X‐100, 20mM HEPES, 140mM NaCl, pH 7.4) containing protease inhibitor cocktail (Sigma). Triton‐insoluble proteins were pelleted by centrifugation, and supernatants were denatured in reducing lithium dodecyl sulfate sample buffer (Thermo Fisher Scientific). Mucin glycoproteins were separated by SDS‐agarose gel electrophoresis as described previously (Warren, Krzesinski, & Greaser, 2003). Briefly, a 1cm 12% polyacrylamide plug was cast using 10 × 8cm glass plates and 1.5mm spacers (Bio‐Rad). After polymerisation, this was overlaid by a 1% agarose gel (Agarose LE, Promega) prepared in running buffer (50mM Tris, 384mM glycine, and 0.1% SDS) containing 30% glycerol. Samples were electrophoresed at 13 mA for 45 min at 4 °C in a Mini‐PROTEAN Tetra Cell device (Bio‐Rad). For electroblotting, gels were placed into NuPAGE transfer buffer (Thermo Fisher Scientific) containing 10% methanol and blotted to 0.45 μm Hybond PVDF membrane (GE Healthcare Life Sciences) at 40 V for 2 hr 20 min at 4 °C in an Xcell II blot module (Thermo Fisher Scientific). Membranes were subsequently blocked with 3% BSA in Tris‐buffered saline/0.05% Tween‐20 for 60 min and incubated with mouse anti‐MUC2 (abcam) overnight at 4 °C. After washing, blots were incubated with horseradish peroxidase‐conjugated goat anti‐mouse IgG (Invitrogen) and developed using enhanced chemiluminescence (Immobilon Western, Millipore) and a FluorChem E Imager (ProteinSimple). Signal intensity was quantified using ImageJ software.

### 4.8 Bacterial mutagenesis and complementation

An *stcE* mutant in strain TUV 93‐0 was generated by Lambda Red recombination (Datsenko & Wanner, 2000). Briefly, the helper plasmid pKD46 carrying the Lambda Red recombinase was electroporated into TUV 93‐0, and recombinant bacteria were selected on LB agar containing 100 μg/ml ampicillin. Subsequently, the kanamycin resistance cassette from plasmid pKD4 was polymerase chain reaction (PCR) amplified using Q5 High Fidelity DNA Polymerase (NEB) and primers M1 (5′‐CCGATGAAATTAAAGTATCTGTCATGTACGATCCTTGCCCCTCTGGCGTGTAGGCTGGAGCTGCTTCG‐3′, Sigma‐Genosys) and M2 (5′‐CCTCATTGACCTAGGTTTACTGAAGTCCAAATACTGTCCCATATGAATATCCT‐3′) flanking the resistance gene and including approximately 40 bp extensions complementary to *stcE*. The resulting PCR product was electroporated into pKD46‐recombinant TUV 93‐0, and Lambda Red recombinase expression was induced with 0.2% arabinose (*w*/*v*). Recombinants were selected on LB plates containing 50 μg/mL kanamycin, and clones were cured from pKD46 by incubation at 43 °C. Mutagenesis of *stcE* was confirmed by sequencing (Eurofins) using primers M1 and M2 and external primers E1 (5′‐GCCCTGAAGCTTGCTGAACGCATCGGTG‐3′) and E2 (5′‐CCTGCGCTCTCCCCTCCGATGATAGTGG‐3′) flanking the site of mutation.

For complementation, the *stcE* gene and its promotor were PCR amplified from the pO157 plasmid of TUV 93‐0 using primers stcE‐F (5′‐CTCTGAGGTGTCTGTTAAACCCG‐3′) and stcE‐R (5′‐AAGTGGCCGCACCGTCTC‐3′). The PCR product was blunt end‐ligated into the low copy‐number vector pACYC184 (NEB) digested with NruI (NEB) to generate pACYC184‐*stcE*. Complementing plasmid with catalytically inactive StcE E447D (Yu, Worrall, & Strynadka, 2012) was created by PCR amplifying *stcE* from pACYC184‐*stcE* using primers pairs stcE‐F/stcE_mut‐R (5′P‐TCATGACTGAACTCATTCCC‐3′) and stcE_mut‐F (5′P‐TGTTGGTCATAATTATGGTCTTGG‐3′)/stcE‐R. The resulting amplicons were mixed in equimolar amounts, ligated, and PCR amplified using primers stcE‐F and stcE‐R. Amplification products were subsequently blunt‐end ligated into NruI‐restricted pACYC184 to generate pACYC184‐*stcE*_E447D_. Plasmids were transformed into TUV 93‐0 *ΔstcE* by electroporation, and recombinant clones were selected on LB agar containing 30 μg/ml chloramphenicol. Successful transformation was confirmed by PCR with primers stcE‐F and stcE‐R and sequencing of the entire cloning region using primers stcE‐F, stcE‐R, NruI‐F (5′‐CCACCAAACGTTTCGGCGAG‐3′), NruI‐R (5′‐TGCCTGGACAGCATGGCCTG‐3′), stcE‐F2 95′‐AAAAGTCTGCTGCTTGTCCG‐3′), stcE‐F3 (5′‐GGAATATTTCCAGACCATTCC‐3′) and stcE‐F4 (5′‐GTGGAATGCAGATACGCAGG‐3′).

### 4.9 Quantitative reverse transcription polymerase chain reaction

Total cellular RNA was extracted using the Promega SV RNA extraction kit according to the manufacturer's instructions. RNA concentration and purity was determined using a NanoDrop ND‐1000 spectrophotometer (Fisher Scientific), and RNA integrity was confirmed by agarose gel electrophoresis. For cDNA synthesis, 1 μg RNA was reverse‐transcribed using qScript cDNA supermix (Quanta BioSciences). Quantitative PCR was carried out using SYBR Green JumpStart Taq ReadyMix (Sigma) and an ABI7500 Taqman lightcycler (Applied BioSciences). Primers were purchased from Sigma‐Genosys, and the following sequences were used for amplification of mucin (MUC2 and MUC5AC) and housekeeping genes (RNA polymerase II polypeptide A‐POLR2A and tyrosine 3‐monooxygenase/tryptophan 5‐monooxygenase activation protein, zeta polypeptide‐YWHAZ): MUC2‐F 5′‐ACTGCACATTCTTCAGCTGC‐3′, MUC2‐R 5′‐ATTCATGAGGACGGTCTTGG‐3′, MUC5AC‐F 5′‐CTGGGGTCCTCATTCAGCAG‐3′, MUC5AC‐R 5′‐CCCGAATTCCATGGGTGTCA‐3′, POLR2A‐F 5′‐GATGGGCAAAAGAGTGGACTT‐3′, POLR2A‐R 5′‐GGGTACTGACTGTTCCCCCT‐3′, YWHAZ‐F 5′‐ACTTTTGGTACATTGTGGCTTCAA‐3′, and YWHAZ‐R 5′‐CCGCCAGGACAAACCAGTAT‐3′. Cycling parameters were as follows: 2 min at 95 °C; 30 s at 95 °C, 30 s at 60°C, 35 s at 72°C (40 cycles); 5 min at 72°C. PCR product specificity was confirmed by melt curve analysis and agarose gel electrophoresis. Relative quantification of gene expression was performed using the comparative cycle threshold (Ct) method. Ct values for genes of interest were normalized using the geometric mean Ct of the two housekeepers. Fold expression levels in treated samples were calculated relative to matched non‐treated controls using the formula 2^−ΔΔCt^.

### 4.10 Statistics

All data are shown as means ± standard error of the mean of three independent experiments performed in duplicate unless indicated otherwise. Statistical analysis was performed using GraphPad Prism software (version 5). Student's *t* test or one‐way analysis of variance with Tukey's multiple comparisons test was used to determine differences between two or multiple groups, respectively. For biopsy data, the nonparametric Kruskal‐Wallis test with Dunn's post‐test was used to analyse multiple groups. A *p* value of <0.05 was considered significant.

## Supporting information

Supplementary Figure 1: Adherence of wild‐type (wt) and adhesin‐deficient EHEC strains to Caco‐2 and LS174T cells after 1 h of infection. Adhesion is expressed as percentage of cell‐bound bacteria relative to the inoculum.

## References

[cmi12717-bib-0001] Bartman, A. E. , Sanderson, S. J. , Ewing, S. L. , Niehans, G. A. , Wiehr, C. L. , Evans, M. K. , & Ho, S. B. (1999). Aberrant expression of MUC5AC and MUC6 gastric mucin genes in colorectal polyps. International Journal of Cancer, 80, 210–218. 9935202 10.1002/(sici)1097-0215(19990118)80:2<210::aid-ijc9>3.0.co;2-u

[cmi12717-bib-0002] Bergstrom, K.S. , Guttman, J.A. , Rumi, M. , Ma, C. , Bouzari, S. , Khan, M.A. *, et al.* (2008). Modulation of intestinal goblet cell function during infection by an attaching and effacing bacterial pathogen. Infection and Immunity 76, 796–811. 17984203 10.1128/IAI.00093-07 PMC2223480

[cmi12717-bib-0003] Bergstrom, K. S. , Kissoon‐Singh, V. , Gibson, D. L. , Ma, C. , Montero, M. , Sham, H. P. , et al. (2010). Muc2 protects against lethal infectious colitis by disassociating pathogenic and commensal bacteria from the colonic mucosa. PLoS Pathogens, 6, e1000902. 10.1371/journal.ppat.1000902 PMC2869315 20485566

[cmi12717-bib-0004] Chong, Y. , Fitzhenry, R. , Heuschkel, R. , Torrente, F. , Frankel, G. , & Phillips, A. D. (2007). Human intestinal tissue tropism in Escherichia coli O157: H7‐initial colonization of terminal ileum and Peyer's patches and minimal colonic adhesion *ex vivo* . Microbiology, 153, 794–802. 17322200 10.1099/mic.0.2006/003178-0

[cmi12717-bib-0005] Croxen, M. A. , Law, R. J. , Scholz, R. , Keeney, K. M. , Wlodarska, M. , & Finlay, B. B. (2013). Recent advances in understanding enteric pathogenic Escherichia coli . Clinical Microbiology Reviews, 26, 822–880. 24092857 10.1128/CMR.00022-13 PMC3811233

[cmi12717-bib-0006] Datsenko, K. A. , & Wanner, B. L. (2000). One‐step inactivation of chromosomal genes in Escherichia coli K‐12 using PCR products. Proc.Natl.Acad.Sci.U.S.A, 97, 6640–6645. 10829079 10.1073/pnas.120163297 PMC18686

[cmi12717-bib-0007] Erdem, A. L. , Avelino, F. , Xicohtencatl‐Cortes, J. , & Girón, J. A. (2007). Host protein binding and adhesive properties of H6 and H7 flagella of attaching and effacing Escherichia coli . J.Bacteriol., 189, 7426–7435. 17693516 10.1128/JB.00464-07 PMC2168434

[cmi12717-bib-0008] Feng, P. C. , Monday, S. R. , Lacher, D. W. , Allison, L. , Siitonen, A. , Keys, C. , et al. (2007). Genetic diversity among clonal lineages within Escherichia coli O157:H7 stepwise evolutionary model. Emerging Infectious Diseases, 13, 1701–1706. 18217554 10.3201/eid1311.070381 PMC3375798

[cmi12717-bib-0009] Fitzhenry, R. J. , Pickard, D. J. , Hartland, E. L. , Reece, S. , Dougan, G. , Phillips, A. D. , & Frankel, G. (2002). Intimin type influences the site of human intestinal mucosal colonisation by enterohaemorrhagic Escherichia coli O157:H7. Gut, 50, 180–185. 11788556 10.1136/gut.50.2.180 PMC1773112

[cmi12717-bib-0010] Fitzhenry, R. , Dahan, S. , Torres, A. G. , Chong, Y. , Heuschkel, R. , Murch, S. H. , et al. (2006). Long polar fimbriae and tissue tropism in Escherichia coli O157:H7. Microbes.Infect., 8, 1741–1749. 16815722 10.1016/j.micinf.2006.02.012

[cmi12717-bib-0011] Freedman, S. B. , Xie, J. , Neufeld, M. S. , Hamilton, W. L. , Hartling, L. , & Tarr, P. I. (2016). Shiga toxin‐producing Escherichia coli infection, antibiotics, and risk of developing hemolytic uremic syndrome: A meta‐analysis. Clinical Infectious Diseases, 62, 1251–1258. 26917812 10.1093/cid/ciw099 PMC4845788

[cmi12717-bib-0012] Gagnon, M. , Zihler Berner, A. , Chervet, N. , Chassard, C. , & Lacroix, C. (2013). Comparison of the Caco‐2, HT‐29 and the mucus‐secreting HT29‐MTX intestinal cell models to investigate *Salmonella* adhesion and invasion. Journal of Microbiological Methods, 94, 274–279. 23835135 10.1016/j.mimet.2013.06.027

[cmi12717-bib-0013] Gould, L. H. , Mody, R. K. , Ong, K. L. , Clogher, P. , Cronquist, A. B. , Garman, K. N. , et al. (2013). Increased recognition of non‐O157 Shiga toxin‐producing Escherichia coli infections in the United States during 2000‐2010: Epidemiologic features and comparison with E. coli O157 infections. Foodborne Pathogens and Disease, 10, 453–460. 23560425 10.1089/fpd.2012.1401

[cmi12717-bib-0014] Grys, T. E. , Siegel, M. B. , Lathem, W. W. , & Welch, R. A. (2005). The StcE protease contributes to intimate adherence of enterohemorrhagic Escherichia coli O157:H7 to host cells. Infection and Immunity, 73, 1295–1303. 15731026 10.1128/IAI.73.3.1295-1303.2005 PMC1064933

[cmi12717-bib-0015] Grys, T. E. , Walters, L. L. , & Welch, R. A. (2006). Characterization of the StcE protease activity of Escherichia coli O157:H7. Journal of Bacteriology, 188, 4646–4653. 16788173 10.1128/JB.01806-05 PMC1482996

[cmi12717-bib-0016] Gustafsson, J. K. , Navabi, N. , Rodriguez‐Piñeiro, A. M. , Alomran, A. H. , Premaratne, P. , Fernandez, H. R. , et al. (2013). Dynamic changes in mucus thickness and ion secretion during *Citrobacter rodentium* infection and clearance. PloS One, 8, e84430. 10.1371/journal.pone.0084430 PMC3875541 24386378

[cmi12717-bib-0017] Henderson, I. R. , Czeczulin, J. , Eslava, C. , Noriega, F. , & Nataro, J. P. (1999). Characterization of pic, a secreted protease of *Shigella flexneri* and enteroaggregative Escherichia coli . Infect.Immun., 67, 5587–5596. 10531204 10.1128/iai.67.11.5587-5596.1999 PMC96930

[cmi12717-bib-0018] Huet, G. , Kim, I. , de Bolos, C. , Lo‐Guidice, J. M. , Moreau, O. , Hemon, B. , et al. (1995). Characterization of mucins and proteoglycans synthesized by a mucin‐secreting HT‐29 cell subpopulation. Journal of Cell Science, 108(Pt 3), 1275–1285. 7622610 10.1242/jcs.108.3.1275

[cmi12717-bib-0019] In, J. , Foulke‐Abel, J. , Zachos, N. C. , Hansen, A. M. , Kaper, J. B. , Bernstein, H. D. , et al. (2016). Enterohemorrhagic Escherichia coli reduces mucus and intermicrovillar bridges in human stem cell‐derived colonoids. Cellular and Molecular Gastroenterology and Hepatology, 2, 48 62.e43. 26855967 10.1016/j.jcmgh.2015.10.001 PMC4740923

[cmi12717-bib-0020] Johansson, M. E. , Phillipson, M. , Petersson, J. , Velcich, A. , Holm, L. , & Hansson, G. C. (2008). The inner of the two Muc2 mucin‐dependent mucus layers in colon is devoid of bacteria. Proceedings of the National Academy of Sciences of the United States of America, 105, 15064–15069. 18806221 10.1073/pnas.0803124105 PMC2567493

[cmi12717-bib-0021] Johansson, M. E. , Gustafsson, J. K. , Holmen‐Larsson, J. , Jabbar, K. S. , Xia, L. , Xu, H. , et al. (2014). Bacteria penetrate the normally impenetrable inner colon mucus layer in both murine colitis models and patients with ulcerative colitis. Gut, 63, 281–291. 23426893 10.1136/gutjnl-2012-303207 PMC3740207

[cmi12717-bib-0022] Juge, N. (2012). Microbial adhesins to gastrointestinal mucus. Trends in Microbiology, 20, 30–39. 22088901 10.1016/j.tim.2011.10.001

[cmi12717-bib-0023] Lathem, W. W. , Grys, T. E. , Witowski, S. E. , Torres, A. G. , Kaper, J. B. , Tarr, P. I. , & Welch, R. A. (2002). StcE, a metalloprotease secreted by Escherichia coli O157:H7, specifically cleaves C1 esterase inhibitor. Molecular Microbiology, 45, 277–288. 12123444 10.1046/j.1365-2958.2002.02997.x

[cmi12717-bib-0024] Lewis, S. B. , Cook, V. , Tighe, R. , & Schüller, S. (2015). Enterohemorrhagic Escherichia coli colonization of human colonic epithelium *in vitro* and *ex vivo* . Infection and Immunity, 83, 942–949. 25534942 10.1128/IAI.02928-14 PMC4333473

[cmi12717-bib-0025] Lindén, S. K. , Florin, T. H. , & McGuckin, M. A. (2008). Mucin dynamics in intestinal bacterial infection. PloS One, 3, e3952. 10.1371/journal.pone.0003952 PMC2601037 19088856

[cmi12717-bib-0026] McGuckin, M. A. , Lindén, S. K. , Sutton, P. , & Florin, T. H. (2011). Mucin dynamics and enteric pathogens. Nature Reviews. Microbiology, 9, 265–278. 21407243 10.1038/nrmicro2538

[cmi12717-bib-0027] McWilliams, B. D. , & Torres, A. G. (2014). Enterohemorrhagic Escherichia coli adhesins. Microbiology spectrum, 2. 10.1128/microbiolspec.EHEC-0003-2013 26103974

[cmi12717-bib-0028] Navabi, N. , McGuckin, M. A. , & Lindén, S. K. (2013). Gastrointestinal cell lines form polarized epithelia with an adherent mucus layer when cultured in semi‐wet interfaces with mechanical stimulation. PloS One, 8, e68761. 10.1371/journal.pone.0068761 PMC3712011 23869232

[cmi12717-bib-0029] Nesta, B. , Valeri, M. , Spagnuolo, A. , Rosini, R. , Mora, M. , Donato, P. , et al. (2014). SslE elicits functional antibodies that impair in vitro mucinase activity and in vivo colonization by both intestinal and extraintestinal Escherichia coli strains. PLoS Pathogens, 10, e1004124. 10.1371/journal.ppat.1004124 PMC4014459 24809621

[cmi12717-bib-0030] Rendón, M. A. , Saldaña, Z. , Erdem, A. L. , Monteiro‐Neto, V. , Vázquez, A. , Kaper, J. B. , et al. (2007). Commensal and pathogenic Escherichia coli use a common pilus adherence factor for epithelial cell colonization. Proceedings of the National Academy of Sciences of the United States of America, 104, 10637–10642. 17563352 10.1073/pnas.0704104104 PMC1890562

[cmi12717-bib-0031] Ritchie, J. M. (2014). Animal models of enterohemorrhagic Escherichia coli infection. Microbiology spectrum, 2, Ehec‐0022‐2013. 10.1128/microbiolspec.EHEC-0022-2013 26104195

[cmi12717-bib-0032] Rossez, Y. , Wolfson, E. B. , Holmes, A. , Gally, D. L. , & Holden, N. J. (2015). Bacterial flagella: Twist and stick, or dodge across the kingdoms. PLoS Pathogens, 11, e1004483. 10.1371/journal.ppat.1004483 PMC4295861 25590430

[cmi12717-bib-0033] Silva, A. J. , Pham, K. , & Benitez, J. A. (2003). Haemagglutinin/protease expression and mucin gel penetration in el tor biotype *Vibrio cholerae* . Microbiology, 149, 1883–1891. 12855739 10.1099/mic.0.26086-0

[cmi12717-bib-0034] Soscia, C. , Hachani, A. , Bernadac, A. , Filloux, A. , & Bleves, S. (2007). Cross talk between type III secretion and flagellar assembly systems in *Pseudomonas aeruginosa* . Journal of Bacteriology, 189, 3124–3132. 17307856 10.1128/JB.01677-06 PMC1855843

[cmi12717-bib-0035] Stevens, M. P. , & Frankel, G. M. (2014). The locus of Enterocyte effacement and associated virulence factors of enterohemorrhagic Escherichia coli . Microbiology spectrum, 2, Ehec‐0007‐2013. 10.1128/microbiolspec.EHEC-0007-2013 26104209

[cmi12717-bib-0036] Szabady, R. L. , Lokuta, M. A. , Walters, K. B. , Huttenlocher, A. , & Welch, R. A. (2009). Modulation of neutrophil function by a secreted mucinase of Escherichia coli O157:H7. PLoS Pathogens, 5, e1000320. 10.1371/journal.ppat.1000320 PMC2642718 19247439

[cmi12717-bib-0037] Szabady, R. L. , Yanta, J. H. , Halladin, D. K. , Schofield, M. J. , & Welch, R. A. (2011). TagA is a secreted protease of *Vibrio cholerae* that specifically cleaves mucin glycoproteins. Microbiology, 157, 516–525. 20966091 10.1099/mic.0.044529-0 PMC3090133

[cmi12717-bib-0038] Trachtman, H. , Austin, C. , Lewinski, M. , & Stahl, R. A. (2012). Renal and neurological involvement in typical Shiga toxin‐associated HUS. Nature Reviews. Nephrology, 8, 658–669. 22986362 10.1038/nrneph.2012.196

[cmi12717-bib-0039] Tzipori, S. , Karch, H. , Wachsmuth, I. K. , Robins‐Browne, R. M. , O'Brien, A. D. , Lior, H. , et al. (1987). Role of a 60‐megadalton plasmid and Shiga‐like toxins in the pathogenesis of enterohaemorrhagic Escherichia coli O157:H7 in gnotobiotic piglets. Infection and Immunity, 55, 3117–3125. 3316033 10.1128/iai.55.12.3117-3125.1987 PMC260036

[cmi12717-bib-0040] Valeri, M. , Rossi Paccani, S. , Kasendra, M. , Nesta, B. , Serino, L. , Pizza, M. , & Soriani, M. (2015). Pathogenic E. coli exploits SslE mucinase activity to translocate through the mucosal barrier and get access to host cells. PloS One, 10, e0117486. 10.1371/journal.pone.0117486 PMC4366376 25789808

[cmi12717-bib-0041] van Klinken, B. J. , Oussoren, E. , Weenink, J. J. , Strous, G. J. , Buller, H. A. , Dekker, J. , & Einerhand, A. W. (1996). The human intestinal cell lines Caco‐2 and LS174T as models to study cell‐type specific mucin expression. Glycoconjugate Journal, 13, 757–768. 8910003 10.1007/BF00702340

[cmi12717-bib-0042] Vieira, M. A. , Gomes, T. A. , Ferreira, A. J. , Knöbl, T. , Servin, A. L. , & Liévin‐Le Moal, V. (2010). Two atypical enteropathogenic Escherichia coli strains induce the production of secreted and membrane‐bound mucins to benefit their own growth at the apical surface of human mucin‐secreting intestinal HT29‐MTX cells. Infection and Immunity, 78, 927–938. 20065027 10.1128/IAI.01115-09 PMC2825950

[cmi12717-bib-0043] Walsham, A. D. , MacKenzie, D. A. , Cook, V. , Wemyss‐Holden, S. , Hews, C. L. , Juge, N. , & Schüller, S. (2016). *Lactobacillus reuteri* inhibition of enteropathogenic Escherichia coli adherence to human intestinal epithelium. Frontiers in Microbiology, 7, 244. 26973622 10.3389/fmicb.2016.00244 PMC4771767

[cmi12717-bib-0044] Warren, C. M. , Krzesinski, P. R. , & Greaser, M. L. (2003). Vertical agarose gel electrophoresis and electroblotting of high‐molecular‐weight proteins. Electrophoresis, 24, 1695–1702. 12783444 10.1002/elps.200305392

[cmi12717-bib-0045] Xue, Y. , Zhang, H. , Wang, H. , Hu, J. , Du, M. , & Zhu, M. J. (2014). Host inflammatory response inhibits Escherichia coli O157:H7 adhesion to gut epithelium through augmentation of mucin expression. Infection and Immunity, 82, 1921–1930. 24566630 10.1128/IAI.01589-13 PMC3993425

[cmi12717-bib-0046] Yu, A. C. Y. , Worrall, L. J. , & Strynadka, N. C. J. (2012). Structural insight into the bacterial mucinase StcE essential to adhesion and immune evasion during Enterohemorrhagic E. coli infection. Structure, 20, 707–717. 22483117 10.1016/j.str.2012.02.015

