# Supplementary figures and images for: The StcE metalloprotease of enterohaemorrhagic Escherichia coli reduces the inner mucus layer and promotes adherence to human colonic epithelium ex vivo
Source: Cell Microbiol. 2017 Feb 15;19(6):e12717. doi: 10.1111/cmi.12717 (PMC5434857; doi:10.1111/cmi.12717)

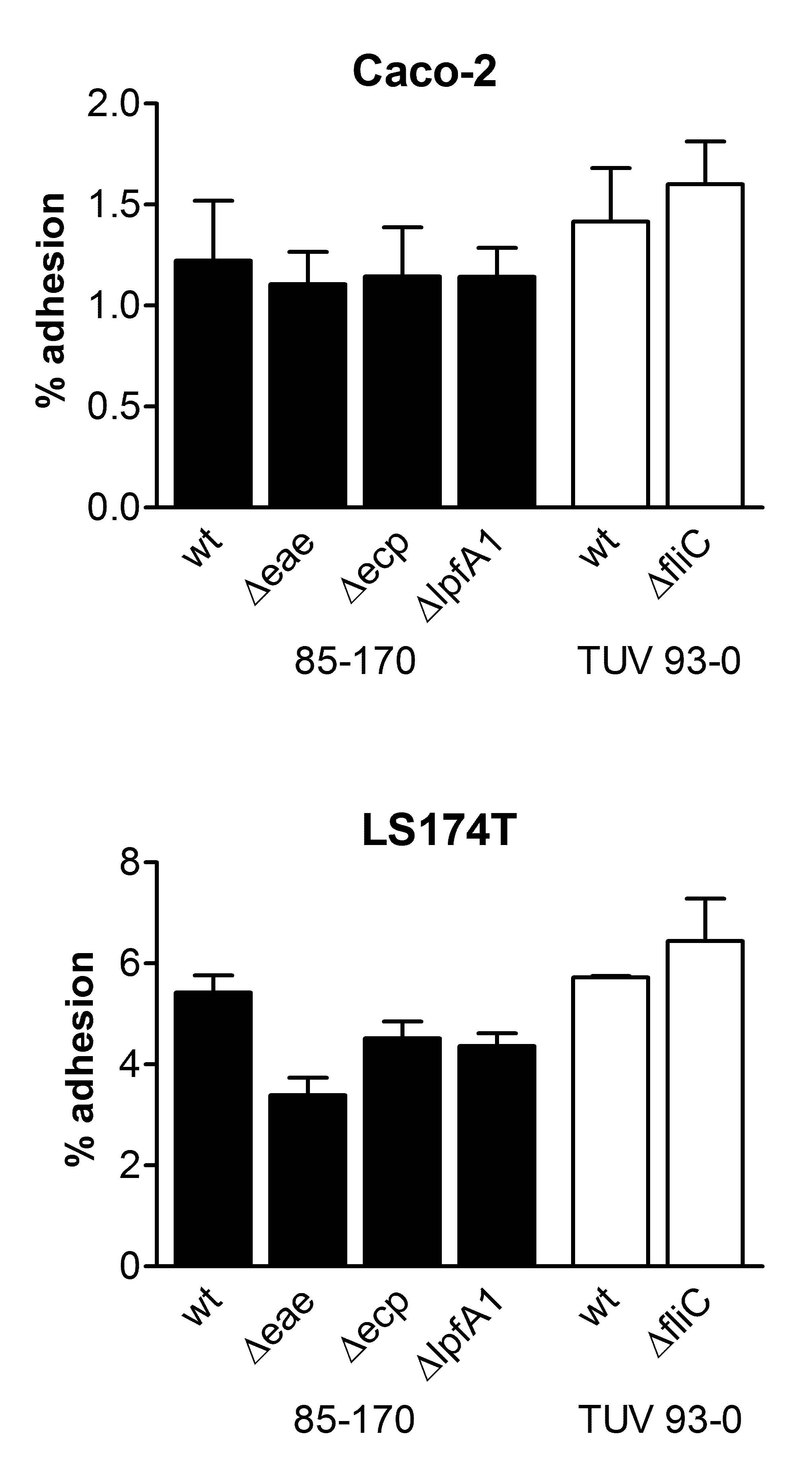

Supplement: Supplementary file 1 — Supplementary Figure 1: Adherence of wild‐type (wt) and adhesin‐deficient EHEC strains to Caco‐2 and LS174T cells after 1 h of infection. Adhesion is expressed as percentage of cell‐bound bacteria relative to the inoculum. [file CMI-19-na-s001.tif]
